# Linkage analysis of anti-CCP levels as dichotomized and quantitative traits using GAW15 single-nucleotide polymorphism scan of NARAC families

**DOI:** 10.1186/1753-6561-1-s1-s107

**Published:** 2007-12-18

**Authors:** Xiaohong R Yang, Kimberly F Kerstann, Andrew W Bergen, Alisa M Goldstein, Lynn R Goldin

**Affiliations:** 1 Division of Cancer Epidemiology and Genetics, National Cancer Institute, 6120 Executive Boulevard, MSC 7236, Bethesda, Maryland 20892, USA

## Abstract

Rheumatoid arthritis is a clinically and genetically heterogeneous disease. Anti-cyclic citrullinated (anti-CCP) antibodies have a high specificity for rheumatoid arthritis and levels correlate with disease severity. The focus of this study was to examine whether analyzing anti-CCP levels could increase the power of linkage analysis by identifying a more homogeneous subset of rheumatoid arthritis patients. We also wanted to compare linkage signals when analyzing anti-CCP levels as dichotomized (CCP_binary), categorical (CCP_cat), and continuous traits, with and without transformation (log_CCP and CCP_cont). Illumina single-nucleotide polymorphism scans of the North American Rheumatoid Arthritis Consortium families were analyzed for four chromosomes (6, 7, 11, 22) using nonparametric linkage (NPL) (rheumatoid arthritis and CCP_binary), *regress *(CCP_cat and Log_CCP), and *deviates *(CCP_cont) analysis options as implemented in Merlin. Similar linkage results were obtained from analyses of rheumatoid arthritis, CCP_binary, and CCP_cont. The only exception was that we observed improved linkage signals and a narrower region for CCP_binary as compared to a clinical diagnosis of rheumatoid arthritis alone on chromosome 7, a region which previously showed variation in linkage results with rheumatoid arthritis according to anti-CCP levels. Analyses of CCP_cat and Log_CCP had little power to detect linkage. Our data suggested that linkage analyses of anti-CCP levels may facilitate identification of rheumatoid arthritis genes but quantitative analyses did not further improve power. Our study also highlighted that quantitative trait linkage results are highly sensitive to phenotype transformation and analytic approaches.

## Introduction

Rheumatoid arthritis (RA) is a chronic inflammatory autoimmune disease affecting about 1% of the population. A genetic component for RA has been well established, with the MHC region being the largest single contributing component. Other chromosome regions (11q, 10q, 14q, 6p, 6q, 16q, 12p, etc.) and candidate genes (*PTPN22*, *CTLA4*, *PADI4*) have been identified by whole-genome linkage scans and association studies [1-5]. Most recently, a high-density SNP analysis of 642 families affected with RA collected by the North American Rheumatoid Arthritis Consortium (NARAC), the largest single linkage study of RA, identified two new linkage regions, 11p and 2q [6]. These findings reflect the genetic complexity of the disease and suggest that analysis of a more homogeneous RA phenotype might increase the power of linkage analysis. In addition, most previous studies have analyzed RA as a dichotomous trait, which could lead to a power loss if RA is a naturally quantitative trait [7].

Anti-cyclic citrullinated (anti-CCP) antibodies have a high specificity for RA [8] and the levels are correlated with disease severity [9,10]. To examine whether the power of linkage analysis could be improved by analyzing a more homogeneous phenotype and by quantitative characterization of the trait, we performed linkage analysis of anti-CCP antibody levels for selected chromosome regions previously linked to RA using NARAC data.

## Methods

### Data set

Illumina SNP scans of the NARAC families were analyzed. We chose anti-CCP antibody levels as the phenotype of interest, and evaluated the effect of covariates including sex, age of onset, year of birth, ever/never smoking, and current smoking. Anti-CCP antibody levels were analyzed in three ways: dichotomized, categorical, and continuous. An antibody titer of 20 was used as a cut-off value to dichotomize anti-CCP levels into positive (>20) and negative (≤20). In addition, anti-CCP levels were characterized into multiple (four) categories (negative, 0–19.9; low, 20–49.9; medium, 50–99.9; high, ≥100). Anti-CCP levels were also analyzed as continuous measurements. Because the assay for anti-CCP antibody titer has an upper limit of 210, we recoded all measurements exceeding 210 to 210. A log transformation was applied to approximate normality of anti-CCP levels because the raw data were highly skewed.

### Chromosome regions

Because the purpose of this study was to compare methods rather than search for a new locus for anti-CCP, we limited our linkage analyses to selected regions. Chromosomes were selected based on findings from a previous SNP scan of NARAC families for RA [6]. Chromosome 6, which contains human lymphocyte antigen (HLA) locus, was chosen as a positive control region. Chromosome 22, which did not show evidence for linkage with RA, was selected as the negative control. Chromosomes 7 and 11, which showed suggestive and significant evidence for linkage with RA, respectively, were included in our linkage analyses as test regions. In particular, it has been shown that chromosome 7 might harbor a susceptibility locus that was more closely linked to anti-CCP positive disease [6].

### Linkage analysis

SNPs on chromosomes 6, 7, 11, and 22 were analyzed for all four anti-CCP phenotypes (CCP_binary, CCP_cat, Log_CCP, and CCP_cont) as well as RA affection status. Linkage disequilibrium (LD) between markers was calculated and markers in LD defined by *D*' > 0.7 were removed using SNPLINK [11]. CCP_binary and RA affection were analyzed by nonparametric (NPL) linkage analysis using Merlin. CCP_cat and Log_CCP were analyzed by regression analysis implemented in Merlin Regress, which uses trait-squared sums and differences to predict IBD sharing between sib pairs [12]. To run Merlin Regress, it was necessary to specify some trait distribution parameters, such as mean, variance, and heritability in the general population. We did not use the sample mean and variance because the families were affected with RA and therefore had higher frequencies and levels of anti-CCP positives. Instead, we estimated the mean (0 for CCP_cat, 0.78 for Log_CCP) and variance (0.0088 for CCP_cat, 0.11 for Log_CCP) among individuals who were anti-CCP negative (≤20) to approximate the distribution in the general population. Heritability of 0.6 was estimated based on variance-component analysis using Merlin. Untransformed continuous anti-CCP levels were also analyzed using the *deviates *option implemented in Merlin, which makes no assumptions about the trait distribution. Again, we chose 0 to approximate the population mean for anti-CCP levels.

## Results

A total of 746 families containing 1794 affected individuals with RA were used for the linkage analyses. There were only 11 individuals in this data set who were unaffected with RA. Anti-CCP data was available for 1499 individuals. Among the 823 sib pairs with available anti-CCP data, there were 77 concordant negative, 228 discordant, and 518 concordant positive for anti-CCP. The distribution of anti-CCP levels was highly skewed toward high positive, with >50% of the individuals having high anti-CCP levels when using cut points suggested by the data provider (Table 1). Male sex and ever smoking were associated with significantly increased anti-CCP values (*t*-test *p *< 0.001 and 0.004, respectively) (Table 2).

**Table 1:** Distribution of anti-CCP levels among the 1499 individuals included in the linkage analyses

CCP category	*N*	%
Negative	341	22.75
Low	167	11.14
Medium	181	12.07
High	810	54.04

**Table 2:** Anti-CCP levels by gender, age, year of birth, and smoking status

Covariate	Values	*N*	anti-CCP mean	SD	t test *P*
Sex	Female	1153	112.19	102.58	<0.001
	Male	346	143.24	100.19	
					
Age of onset	<40	758	119.70	97.53	
	≥40	722	119.89	108.01	ns^a^
					
Year of birth	<1944	754	119.34	97.53	
	≥1944	738	119.50	108.01	ns
					
Smoking	Never	618	110.54	100.38	
	Ever	832	126.13	104.91	0.004
	Current	244	119.84	105.32	
	Never or former	1211	119.44	102.79	ns

^a^ns, not significant

**Figure 1:**
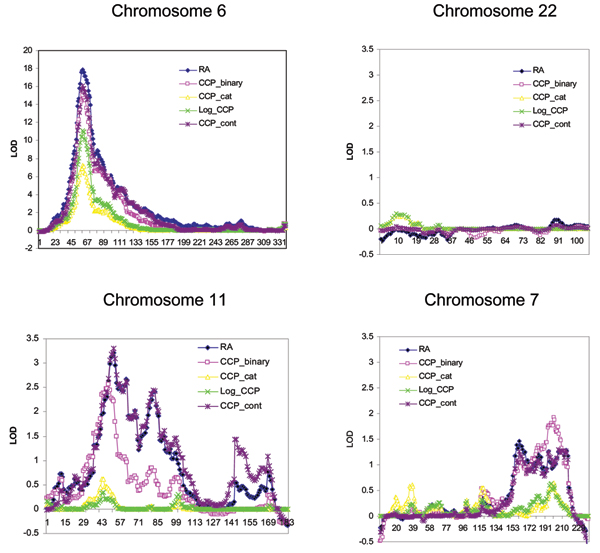
**Linkage analyses of 4 chromosomes for RA and anti-CCP levels using SNPs**. LOD scores for binary outcomes (RA, CCP_binary), transformed quantitative traits (CCP_cat, Log_CCP), and untransformed quantitative trait (CCP_cont) were calculated using Merlin NPL, Merlin *Regress*, Merlin --*deviates*, respectively. The scale of the Y-axis for chromosome 6 is different.

Results obtained from NPL analysis of RA are consistent with those shown previously by Amos et al. [6]. Overall, similar linkage signals were obtained from analyses with RA and CCP_binary, with weaker evidence for linkage with CCP_binary on chromosomes 6 and 11 (Table 3, Figure 1). On the other hand, higher LOD scores and improved definition of a linkage peak for CCP_binary were observed on chromosome 7 (Figure 1), which has been suggested to be more closely linked to anti-CCP compared to RA. Among quantitative anti-CCP levels, only analysis of untransformed anti-CCP levels showed comparable results with that of RA. Analyses of either categorical or log-transformed anti-CCP levels resulted in significantly reduced linkage signals for both positive control (chromosome 6) and test regions (chromosomes 7 and 11). Adjustment of gender and ever smoking in the regression-based linkage analyses did not significantly change the results (data not shown). As expected, chromosome 22 did not show evidence for linkage to any of the phenotypes.

**Table 3:** Maximal LOD scores for RA and anti-CCP phenotypes in the four chromosome regions

Chromosome	RA	CCP binary	CCP categorical	Log CCP	CCP continuous
6	17.86	15.01	7.26	11.10	15.77
7	1.46	1.93	0.65	0.64	1.30
11	3.22	2.56	0.62	0.34	3.30
22	0.08	0.05	0.29	0.31	0.08

## Discussion

In this study, we analyzed anti-CCP levels as dichotomized, categorical, and continuous traits in linkage analyses. On chromosome 7, we observed improved linkage signals and a narrower linkage peak for dichotomized anti-CCP levels as compared to a clinical diagnosis of RA alone, although neither analysis reached statistical significance. Chromosome 7 previously showed stronger evidence for linkage for RA with positive anti-CCP [6]. However, for the three chromosomes we selected that demonstrated evidence for linkage with RA, analyzing anti-CCP levels as quantitative traits did not further improve power to detect linkage compared to RA. In addition, categorization and log transformation of the continuous phenotype resulted in significantly reduced power to detect linkage.

Mapping susceptibility genes is challenging for clinically and genetically heterogeneous diseases such as RA. Examining a more homogenous disease phenotype might increase the power of linkage analysis. Anti-CCP is highly specific for RA and its levels are correlated with disease severity [9,10]. A previous study suggested genetic variability according to anti-CCP status on chromosomes 4, 5, 6, and 7 [6]. Our data on chromosome 7 supports the hypothesis that anti-CCP status might represent a more genetically homogeneous phenotype of RA. In addition, although linkage signals on chromosome 11 were less significant for the CCP_binary phenotype than RA, linkage peak regions on both chromosomes 7 and 11 were narrower, suggesting that analyzing anti-CCP levels as a dichotomous phenotype might be helpful in narrowing candidate regions in fine mapping.

For naturally occurring continuous or polychotomous traits, dichotomization could lead to power loss for linkage analysis [7]. In particular, if the underlying gene confers not only disease susceptibility but also disease severity, treating disease phenotypes as quantitative traits could provide additional information in linkage analysis. We hypothesized that performing quantitative linkage analyses might increase the power to detect linkage for anti-CCP levels. However, analyses of none of the three quantitative phenotypes provided improved linkage signals compared to NPL analysis of RA. In particular, regression-based quantitative linkage analyses of log-transformed or ordinal anti-CCP levels, with or without covariate adjustment, appeared to have reduced power to detect linkage compared to NPL analysis of dichotomized anti-CCP status. We also used the variance components and quantitative trait locus analysis options implemented in Merlin for CCP_cat and Log_CCP phenotypes and observed similar results. For this particular trait, transforming the phenotype to achieve normality reduced the signal due to the major locus. Alternate approaches to handle non-normality are needed for quantitative trait linkage analysis in selected samples. Consistent with another report [13], we found loss of power when treating CCP_cat as continuous. Novel approaches, such as the recently proposed proportional odds latent variable model by Feng et al. [14], need to be further explored. Finally, all these quantitative linkage analyses are very sensitive to the specification of population parameters, such as population mean and variance, chosen for the *regress *and *deviates *analyses. Replacement of the estimated population mean by the sample mean or default values set by the programs resulted in completely different LOD scores. These results further highlight the importance of precise estimates and appropriate analytical approaches in linkage analysis of quantitative traits.

## Conclusion

Linkage analyses of anti-CCP levels may facilitate identification of RA genes by making the phenotype more homogeneous. However, quantitative analyses of anti-CCP levels did not improve power to detect linkage over analysis of RA. Furthermore, log transformation and categorization of anti-CCP levels resulted in reduced power to detect linkage.

## Competing interests

The author(s) declare that they have no competing interests.
